# The effect of different obturation techniques on post-obturation pain and sealer extrusion in single-visit root canal treatment: a prospective clinical randomized study

**DOI:** 10.1007/s00784-025-06487-y

**Published:** 2025-09-11

**Authors:** Hisham Mahmoud Hamdy Abada, Mohamed Abd El Rahman El Shreif, Faten Mohamed Ahmed Ghonimy, Ebtesam Osama Abo El-Mal, Dana Saeed Abd Elmonem El gemaie

**Affiliations:** 1 Department of Endodontics, Faculty of Dentistry, Kafrelsheikh University, Kafrelsheikh, Egypt; 2 Department of Endodontics, Faculty of Dentistry, Menoufia University, Menoufia, Egypt; 3 Department of Restorative Dental Sciences- Endodontic Division, Faculty of Dentistry, Ahram Canadian University, Giza, Egypt; 4 Department of Endodontics, Faculty of Dentistry, Galala University, Suez, Egypt

**Keywords:** Root canal treatment (RCT), Postoperative pain, Single-visit treatment, Bioceramic sealer

## Abstract

**Objectives:**

Postoperative pain remains a significant concern in endodontics. The main aim of this clinical trial was to assess the impact of various obturation technique and sealer types on post-obturation pain and sealer extrusion in single-visit nonsurgical root canal treatments.

**Materials and methods:**

Study participants were recruited through consecutive sampling from patients referred to the Endodontic Department, Faculty of Dentistry, Institution University, diagnosed as asymptomatic irreversible pulpitis. The study was conducted on 150 cases that were equally and randomly allocated to each of the studied groups (30 for each group) as following; Group 1 - cold lateral compaction with CeraSeal (CLC-CS); Group 2 - continuous wave compaction with CeraSeal (CWC-CS); Group 3 - single cone technique with CeraSeal (SC-CS); Group 4 - Cold lateral compaction with AH Plus (CLC-AH); and Group 5 - continuous wave compaction with AH Plus (CWC-AH). All endodontic procedures were performed by a single experienced endodontist to standardize treatment protocols. The main outcomes were the post-obturation pain which was assessed using a visual analog scale (VAS) scale (0–10), and the incidence of sealer extrusion among the studied groups.

**Results:**

At 6, 24, and 72 h the postobturation pain score did not differ significantly between all studied groups. After 12 and 48 h CWC-CS and CLC-CS showed the lowest significant postobturation pain score respectively, and CWC-AH had the most significant postobturation pain score. During the study, none of the patients needed an emergency visit, the pain score was ranged from (0:1.4). Within the same group, the pain intensity began to significantly decrease for gp1 (CLC-CS) after 24 h, for gp3, gp4, and gp5 (SC-CS, CLC-AH, and CWC-AH, respectively) after 12 h, and for gp2 (CWC-CS) after 6 h. After 72 h, the level of pain in each group was around 0. Regardless of the type of sealer used, no significant difference was found between the type of obturation technique and the postoperative pain intensity (*p* = 0.124), while regardless of the type of obturation technique used, there was a significant difference in postoperative pain intensity and sealer type (*p* < 0.001). The incidence of sealer extrusion did not differ significantly between the tested groups (*p* = 0.499), the results showed that the presence of sealer extrusion was associated with higher significant pain score (*p* < 0.001).

**Conclusion:**

The postoperative pain score after Single-Visit RCT for patient diagnosed as asymptomatic irreversible pulpitis was low to moderate pain score, presence of sealer extrusion increased the postoperative pain intensity. Regardless the type of sealer, different obturation techniques did not associated with postoperative pain intensity, while regardless the obturation technique, AH Plus sealer was associated with higher postoperative pain intensity than CeraSeal sealer. Postoperative pain was related to presence of sealer extrusion.

## Introduction

Root canal treatment (RCT) is a vital dental procedure aimed at disinfecting, filling, and preserving teeth that are at risk of extraction due to pulp infection or damage [1]. The primary objective of RCT is to eliminate bacteria from the infected root canal, thereby preventing reinfection and allowing for the preservation of the natural tooth structure. Recent advancements in techniques, procedures, and materials have significantly enhanced the efficacy and safety of RCT, enabling many cases to be completed in a Single-Visit rather than requiring multiple appointments. This shift is not only more convenient for patients, but also reduces the overall duration of treatment [2]. Despite these advancements, postoperative discomfort remains a significant concern in endodontics. Studies have reported varying rates of postoperative pain, ranging from 3 to 58%, indicating that a substantial number of patients experience discomfort following RCT [2]. This variability can be attributed to several factors, including individual patient responses, the complexity of the root canal system, the specific techniques employed during treatment, mechanical trauma from instrumentation, chemical irritation from irrigants and sealers, and microbiological factors all play critical roles in the onset of post-treatment pain [3, 4]. Among the various intra-treatment parameters influencing postoperative discomfort, the choice of endodontic root canal sealer has emerged as a crucial factor. Sealers are essential for filling voids within canals and providing an apical seal to prevent bacterial infiltration. The interaction between sealers and surrounding tissues can provoke an inflammatory response that exacerbates postoperative discomfort. Recent studies emphasize the importance of selecting appropriate sealing materials to minimize adverse effects. For instance, traditional sealers like zinc oxide-eugenol have been widely used due to their favorable properties; however, newer materials such as bioceramic sealers are gaining traction for their superior biocompatibility and sealing ability [5]. The choice of sealer not only affects the immediate outcome of RCT but also has implications for long-term tooth survival. Bioceramic sealers (BS), also known as calcium silicate-based endodontic sealers, have gained prominence in modern dentistry due to their exceptional biocompatibility and intrinsic osteoinductive properties, these characteristics are particularly valuable in decreasing the inflammatory responses that can occur in cases of overfilling during root canal procedures [5].

The success of endodontic treatments heavily relies on the thorough three-dimensional (3D) cleansing, shaping, and obturation of the complex root canal system. This complex process underlines the critical role that endodontic sealers play in conjunction with various root canal obturation techniques, as they significantly influence the manifestation of postoperative pain. For instance, cold lateral compaction (CLC) remains the predominant technique for root canal obturation, primarily due to its simplicity, reliability, cost-effectiveness, and ease of length control management and retreatment. However, this technique is not without its drawbacks. CLC can be labor-intensive and often fails to adequately penetrate irregularities within the root canal anatomy, this limitation can lead to voids in the obturation mass, which may compromise the sealing ability [6]. In contrast, continuous warm compaction (CWC) methods offer distinct advantages over CLC. These techniques allow for a more homogeneous mass of obturation material that conforms more effectively to the anatomical complexities of the root canal system by enhancing the adaptation of the filling material to irregularities and minimizing voids [6]. Recently, the improvement of rotary instrumentation equipment has facilitated the application of the single-cone obturation approach that recommended to use with bioceramic sealers [6]. Several studies have explored the relationship between sealer type and postoperative pain, some revealing that different sealers can elicit varying degrees of tissue response, thereby affecting pain perception after endodontic treatment, while other studies suggest no significant difference in postoperative pain between bioceramic and traditional sealers like AH Plus or zinc oxide-eugenol (ZOE), others highlight the need for further investigation into how these materials interact with periapical tissues [2]. Hence, the objective of this prospective randomized clinical trial is to assess the impact of various sealer type and obturation technique on postoperative pain and sealer extrusion in single-visit nonsurgical root canal treatments. The null hypothesis was that there is no significant difference between the studied groups.

## Materials and methods

### Study design and sample size calculation

**Fig. 1:**
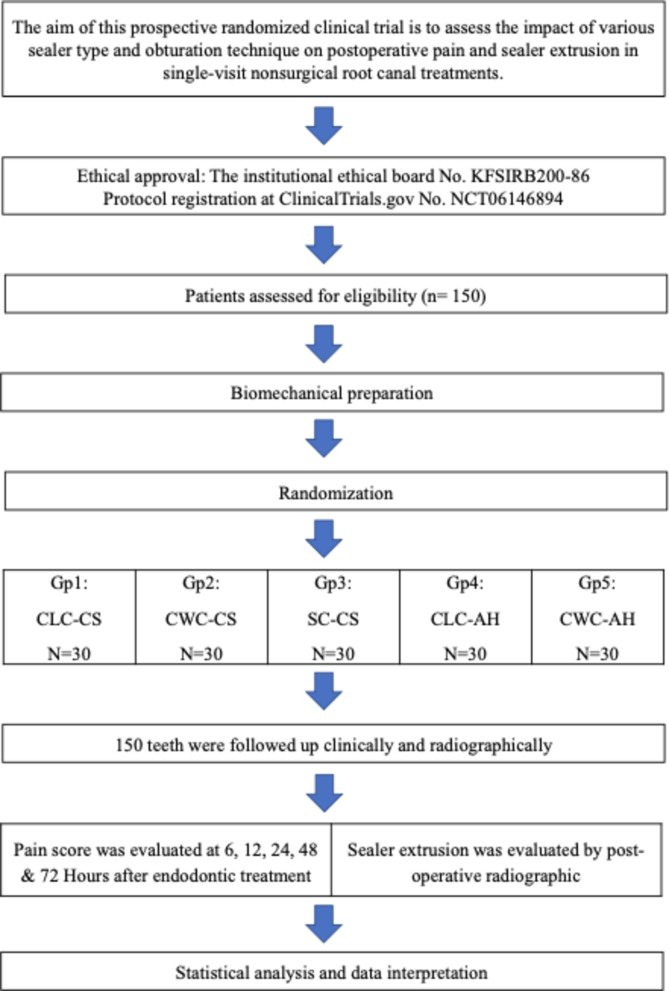
Flowchart presenting the study design

This prospective, randomized, clinical trial was conducted following approval by the institutional ethical board and the study protocol was registered at ClinicalTrials.gov (No. NCT06146894 at https://register.clinicaltrials.gov) and followed the CONSORT guidelines for randomized clinical trials [7]. Study participants were recruited through consecutive sampling from patients referred to the Endodontic Department at the institutional University between December 2023 to June 2024. Based on information from a previous research study [8], power analysis was calculated using G*Power software based on type I error of 0.05 and the power of 80%. A minimum of 27 patients per group was required for the sample size. 30 patients were included in each group to enhance the study’s statistical power and compensate for any patient loss. Figures 1 showing a flowchart presenting the study design.

### Inclusion and exclusion criteria

Systemically healthy patients (American Society of Anesthesiologists Physical Status Classification I or II) aged 18 to 60 years who required nonsurgical root canal treatment for a mandibular lower first molar tooth diagnosed as asymptomatic irreversible pulpitis in which the patient has no clinical symptoms and there was a deep caries lesion extending to the pulp space on the periapical radiographic film [9], three digital periapical radiographs taken (1 straight and 2 angled) to select teeth with three separate root canals, the diagnosis of asymptomatic irreversible pulpitis was confirmed by no pain with percussion test and there was delayed positive response to thermal sensibility and electric pulp tests, and during caries excavation there was a pulpal perforation in which bleeding did not stop after five minutes applying a piece of cotton soaked with NaOCL as a hemostatic agent. Additionally, all teeth were required to be periodontally healthy with probing depths ≤ 3 mm.

Patients were not included if they presented with diabetes mellitus, immunocompromised status, pregnancy, or lactation, and if they had taken antibiotics, analgesics, or anti-inflammatory medications within seven days of beginning endodontic therapy. Further exclusion criteria included necrotic or symptomatic pulp, presence of calcified canal or root resorption, non-restorable teeth, previous initiated root canal therapy, previous root canal treatment, teeth showed radiographic evidence of periapical pathosis (PAI score > 2) [10], immature root, teeth displayed root fractures or cracks identified through transillumination, and teeth exhibited periodontal involvement (mobility > grade 1).

All patient who participated in this study received written informed consent after giving a verbal and written explanation of the objective, clinical procedure, and possible complications. Written informed consent was obtained from all participants following the Declaration of Helsinki guidelines [11]. The digital periapical radiographs were obtained using a paralleling technique with a sensor positioning device (Kodak RVG 5100; Eastman Kodak Company, Rochester, NY, USA) at standardized exposure parameters (70 kV, 7 mA, 0.12 s) and saved in TIFF format for future analysis.

### Randomization

150 patients meeting the inclusion criteria were randomly allocated using a computer-generated sequence (Random Allocation Software version 2.0) into five groups (*n* = 30 each) based on the obturation technique and sealer type. The groups were: Group 1 - Cold lateral compaction with CeraSeal (CLC-CS) (META BIOMED CO., LTD, Korea); Group 2 - Continuous wave compaction with CeraSeal (CWC-CS); Group 3 - Single cone technique with CeraSeal (SC-CS); Group 4 - cold lateral compaction with AH Plus (CLC-AH) (Dentsply DeTreY GmbH, Konstanz, Germany); and Group 5 - continuous wave compaction with AH Plus (CWC-AH). A research assistant concealed allocations by utilizing sealed envelopes that were unsealed immediately before the interventions, the operator was blinded to the nature of intervention (obturation technique and the type of sealer) until finishing the biomechanical preparation.

### Clinical procedure

All endodontic procedures were performed by a single experienced endodontist (H.A.) for all eligible patient to standardize treatment protocols. Local anesthesia was administered using 1.7 mL of 4% articaine hydrochloride with 1:100,000 epinephrine (Inibsa Dental S.L.U., Barcelona, Spain) via an inferior alveolar nerve block. Caries was removed under rubber dam isolation (Hygienic; Coltene/Whaledent Inc, Cuyahoga Falls, OH) using a new sterile #2 round diamond at high speed with water cooling, a hemostatic agent was applied to the pulpal perforation site for five minutes, and if the bleeding did not stop after that, complete deroofing of the pulp chamber roof was performed using an Endo-Z bur (Dentsply Maillefer) at high speed with water cooling, the coronal pulp tissue was removed using sharp excavator and the pulp chamber was irrigated by 5.25% sodium hypochlorite (NaOCL), and the canal orifices were located under 3.5× magnification loupes with LED headlight.

Pre-endodontic build up was performed when needed prior to accessing the root canals, initial canal patency was established using #10 and #15 K-files (Mani Inc., Japan), root canals were scouted, and any teeth did not have three separate root canals were excluded from the study, the working length was determined using an electronic apex locator (Tri Auto-ZX; J Morita USA, Inc, Irvine, CA) set to 0.5 mm short of the apex “0.0” reading. Working length was confirmed radiographically using a paralleling technique. Root canal shaping was performed using ProTaper Next rotary files (Dentsply Maillefer, Ballaigues, Switzerland) following the manufacturer’s recommended sequence. The mesial canals were prepared to X2 (25/0.06), while distal canals were prepared to X3 (30/0.06). New set of files were used for each patient, throughout instrumentation, canals were irrigated with 2 mL of 5.25% NaOCl between each file using a 30-gauge side-vented needle (NaviTip; Ultradent, South Jordan, UT) positioned 1 mm short of working length. The final irrigation protocol consisted of 2 mL of 17% EDTA (Largal Ultra; Septodont, Saint-Maur-des-Fosses, France) for 60 s, followed by 5 mL of sterile saline. Canals were dried with size-matched sterile paper points (Dentsply Maillefer) tell the canals become completely dry in cases of using AH Plus sealer, while in cases of using CeraSeal sealer the canals were kept slightly wet, which could be obtained by stopping dryness when the 3–4 mm from the paper point tip was wet [12]. Obturation was performed according to group allocation using either CeraSeal or AH Plus sealers, prepared and manipulated according to manufacturer’s instructions, and using one of the tested obturation technique (CLC, CWC, and SC technique). For CLC technique, the size-matched gutta-percha master cones were fitted to working length and verified radiographically. Finger spreader (Dentsply Maillefer) with stop set 1 mm short of the working length was used to create space alongside the gutta-percha master cone, accessory cones were used to fill the root canal space and laterally compacted using the finger spreader until the obturation was completed. For CWC technique, System B (SybronEndo, Orange, CA) was set at 200 °C for down-packing, obtura spartan plugger was selected that bind the root canal approximately 5 mm short of WL, and Obtura III (Obtura Spartan, Earth City, MO) at 160 °C for backfilling. The SC technique utilized size-matched gutta-percha points without additional gutta-percha points, the coronal part of the master gutta-percha cone was condensed with gentle pressure using an endodontic plugger. Access cavities were temporarily sealed with glass ionomer cement (Fuji IX GP; GC Corporation, Tokyo, Japan), and immediate postoperative radiographs were taken using standardized exposure parameters same as that used for preoperative exposure.

### Evaluation criteria

Post-obturation pain was assessed using visual analog scale (VAS) scale (0–10), the scale was thoroughly explained to each patient where 0 showed no pain, 1–3 mild pain, 4–6 moderate pain, 7–9 severe pain, and 10 worst possible pain. Patients were provided with standardized VAS forms instructed to record their pain levels at specific time intervals: 6, 12-, 24-, 48-, and 72-hours post-treatment. To ensure accurate reporting, patients received automated SMS reminders at each evaluation time point, and completed forms were collected during the follow-up visit.

Radiographic assessment of sealer extrusion was conducted using standardized digital periapical radiographs taken immediately after obturation. Images were exported in TIFF format and processed using Adobe Photoshop CS6 (Adobe Systems Inc., San Jose, CA, USA) to standardize contrast and brightness. Two calibrated endodontists (M.E. and D.E.) with a minimum of 10 years of clinical experience, who were not involved in the treatment procedures, independently evaluated the radiographs. Before evaluation, the examiners underwent calibration sessions using a set of 20 reference radiographs until achieving an inter-examiner Cohen’s kappa coefficient of > 0.8. Sealer extrusion was recorded as “present” when radiopaque material was detected beyond the radiographic apex compared to baseline radiographs, or “absent” when all filling materials were confined within the root canal space, cases presenting an extrusion > 2 mm of the endodontic sealer were excluded from the study. In cases of disagreement, a third experienced endodontist was consulted to reach a consensus.

### Statistical analysis

Categorical data were presented as frequency and percentage values and were analyzed using chi square test. Numerical data was represented as mean, standard deviation (SD), median and interquartile range (IQR) values. They were analyzed for normality by checking data distribution and by using Shapiro-Wilk’s test. Pain score data were non-parametric and were analyzed using Kruskal-Wallis’s test followed by Dunn’s post hoc test for intergroup comparisons and different associations. Additionally, they were analyzed using Freidman’s test followed by Nemenyi post hoc test for intragroup comparisons. Age data were normally distributed and were analyzed using one-way ANOVA test. P-values were adjusted for multiple comparisons using the False Discovery Rate (FDR) method. The significance level was set at *p* < 0.05 within all tests. Statistical analysis was performed with R statistical analysis software version 4.4.2 for Windows^1^.

## Results

The study was conducted on 150 cases that were equally and randomly allocated to each of the studied groups (i.e., 30 cases each). There was no statistically significant difference between tested groups regarding different age and gender distribution (*p* > 0.05). Demographic characteristics are presented in Table 1.

**Table 1:** Demographic data

Parameter		CLC-CS	CWC-CS	SC-CS	CLC-AH	CWC-AH	Test statistic	*p*-value
**Gender**	**Male**	12 (40%)	15 (50%)	12 (40%)	12 (40%)	9 (30%)	**2.50**	**0.645**
	**Female**	18 (60%)	15 (50%)	18 (60%)	18 (60%)	21 (70%)		
**Age (years)**	**Mean ± SD**	30.10 ± 4.95	28.40 ± 6.47	28.30 ± 5.48	28.50 ± 6.15	30.60 ± 8.37	**0.29**	**0.884**

**Fig. 2:**
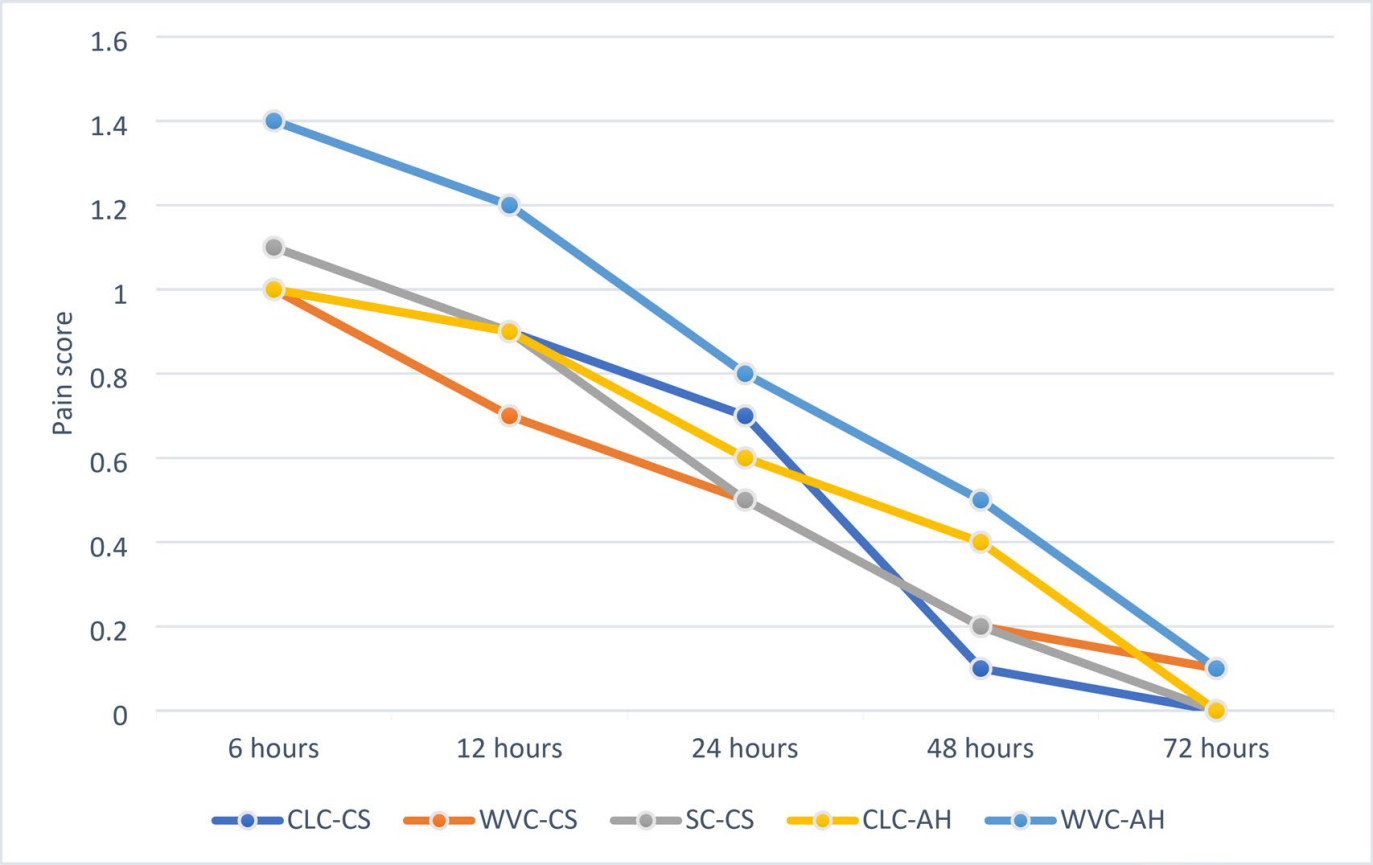
Line chart showing average pain scores

The mean and standard deviation of the pain score measured for all groups at different time interval are presented in Table 2 and in figures 2. There was no significant difference in the postobturation pain results between all tested groups at 6, 24, and 72 h (*p* = 0.068, 0.171 and 0.068 respectively), after 12 h (*p* = 0.018) CWC-CS showed the lowest significant postobturation pain score comparing to other groups. After 48 h (*p* = 0.015) CLC-CS showed the lowest significant postobturation pain score. The CWC-AH showed the highest significant postobturation pain score after 12 and 48 h.

**Table 2:** Summary statistics, inter and intragroup comparisons of pain score for all groups at different time interval

Interval	Measurement	CLC-CS	CWC-CS	SC-CS	CLC-AH	CWC-AH	Test statistic	*p*-value
*6 h*	Mean ± SD	1.00 ± 0.79^Aa^	1.00 ± 0.64^Aa^	1.10 ± 0.55^Aa^	1.00 ± 0.45^Aa^	1.40 ± 0.50^Aa^	9.47	0.068
	Median (IQR)	1.00 (2.00)^Aa^	1.00 (0.00)^Aa^	1.00 (0.00)^Aa^	1.00 (0.00)^Aa^	1.00 (0.00)^Aa^		
*12 h*	Mean ± SD	0.90 ± 0.71^ABa^	0.70 ± 0.47^Bb^	0.90 ± 0.55^ABa^	0.90 ± 0.31^ABa^	1.20 ± 0.41^Aa^	13.95	0.018*
	Median (IQR)	1.00 (1.00)^ABa^	1.00 (1.00)^Ab^	1.00 (0.00)^ABa^	1.00 (0.00)^ABa^	1.00 (0.00)^Ba^		
*24 h*	Mean ± SD	0.70 ± 0.47^Aa^	0.50 ± 0.51^Ac^	0.50 ± 0.51^Ab^	0.60 ± 0.50^Ab^	0.80 ± 0.61^Ab^	6.40	0.171
	Median (IQR)	1.00 (1.00)^Aa^	0.50 (1.00)^Ac^	0.50 (1.00)^Ab^	1.00 (1.00)^Ab^	1.00 (1.00)^Ab^		
*48 h*	Mean ± SD	0.10 ± 0.31^Cb^	0.20 ± 0.41^BCd^	0.20 ± 0.41^BCc^	0.40 ± 0.50^ABc^	0.50 ± 0.51^Ac^	15.96	0.015*
	Median (IQR)	0.00 (0.00)^Bb^	0.00 (0.00)^BCd^	0.00 (0.00)^BCc^	0.00 (1.00)^ACc^	0.50 (1.00)^Ac^		
*72 h*	Mean ± SD	0.00 ± 0.00^Ab^	0.10 ± 0.31^Ad^	0.00 ± 0.00^Ac^	0.00 ± 0.00^Ad^	0.10 ± 0.31^Ad^	9.31	0.068
	Median (IQR)	0.00 (0.00)^Ab^	0.00 (0.00)^Ad^	0.00 (0.00)^Ac^	0.00 (0.00)^Ad^	0.00 (0.00)^Ad^		
*Test statistic*		66.66	68.22	67.42	62.55	82.25		
*p-value*		< 0.001*	< 0.001*	< 0.001*	< 0.001*	< 0.001*		

Values with **different upper and lowercase superscripts** within the **same horizontal row and vertical column**, respectively, are significantly different

* significant (*p* < 0.05)

All patients did not require emergency visit during the study, the pain intensity was found to be no pain score (0) to mild pain score (1:3) ranged from (0:1.4), within the same group there was a significant difference observed through the time interval (*p* < 0.001), the pain intensity started to decrease significantly for gp1(CLC-CS) after 24 h, for gp3, gp4 and gp5 (SC-CS, CLC-AH and CWC-AH respectively) after 12 h, while for gp2 (CWC-CS) after 6 h. For all groups, after 72 h, the pain intensity for all groups was around 0.

Summary statistics and results of intergroup comparison, obturation technique, and sealer type for the incidence of sealer extrusion are presented in Table 3, there was no significant difference in the incidence of sealer extrusion detected between tested groups (*p* = 0.499), the highest incidence of sealer extrusion was detected with SC-CS (60%), and the lowest incidence was associated with CLC-CS (40%), the CWC group showed 50% incidence of sealer extrusion. Neither the obturation technique used or the type of the sealer was associated with higher incidence of sealer extrusion (*p* = 0.168 and 0.548 respectively), using CWC obturation technique was associated with 50% incidence of sealer extrusion, while CLC was associated with 40% incidence of sealer extrusion regardless the type of sealer used, the incidence of sealer extrusion with using CS was 50% while for AH plus was 45%. The results for the incidence of sealer extrusion of the two used sealer type**s** (CS and AH Plus) across different obturation technique showed that there was no significant difference in the incidence of sealer extrusion regarding the use of different obturation technique with the two types of sealer used independently (*p* = 0.436 for CS and AH Plus), regarding the effect of sealer type with different obturation technique independently, both type of sealer showed the same percentage of sealer extrusion with the CLC and CWC technique (40% and 50% for both sealer type with CLC and CWC), and CS showed 60% incidence of sealer extrusion with SC technique.

**Table 3:** Association between sealer extrusion with intergroup comparisons, obturation technique, and sealer type

**Association between sealer extrusion and intergroup comparisons.**							
Sealer extrusion	n (%)					Test statistic	p-value
	CLC-CS	CWC-CS	SC-CS	CLC-AH	CWC-AH		
No	18 (60%)	15 (50%)	12 (40%)	18 (60%)	15 (50%)	3.37	0.499
Yes	12 (40%)	15 (50%)	18 (60%)	12 (40%)	15 (50%)		
**Association between sealer extrusion and obturation technique**							
Sealer extrusion	n (%)					Test statistic	p-value
	CLC		CWC	SC			
No	36 (60%)		30 (50%)	12 (40%)		3.37	0.186
Yes	24 (40%)		30 (50%)	18 (60%)			
**Association between sealer extrusion and sealer type.**							
Sealer extrusion	n (%)					Test statistic	p-value
	CS		AH Plus				
No	45 (50%)		33 (55%)			0.36	0.548
Yes	45 (50%)		27 (45%)				
**Association between sealer extrusion of the two used sealer types across different obturation techniques**							
Sealer	Sealer extrusion	n (%)				Test statistic	p-value
		CLC	CWC	SC			
CS	No	18 (60%)	15 (50%)	12 (40%)		2.40	0.436
	Yes	12 (40%)	15 (50%)	18 (60%)			
AH Plus	No	18 (60%)	15 (50%)	NA		0.61	0.436
	Yes	12 (40%)	15 (50%)	NA			
Test statistic		0.00	0.00	NA			
p-value		1	1	NA			

NA Not Applicable

Table 4 showing associations between pain score and gender, obturation technique, sealer type, and presence of sealer extrusion. The results showed that the female patient was associated with higher significant difference pain score over male patient (*p* < 0.001), there was no significant difference detected between the pain intensity and type of obturation technique regardless the type of sealer used (*p* = 0.124), and when using CS sealer (*p* = 0.89), while using AH plus sealer the pain intensity was significantly higher using CWC technique than using CLC technique (*p* = 0.01). There was a significant difference detected between the pain intensity and type of sealer used regardless the type of obturation technique (*p* < 0.001), and when using CLC technique (*p* = 0.33), using CWC technique the AH plus sealer was associated with significantly higher pain intensity than using CS sealer (*p* < 0.001). Results presented that presence of sealer extrusion was associated with higher significant pain score regardless the type of sealer used (*p* < 0.001), and with each type of sealer independently (*p* = 0.004 and 0.03 for CS and AH Plus respectively).

**Table 4:** Associations between pain score with gender, obturation technique, sealer type, and presence of sealer extrusion

**Associations between pain and gender.**						
Measurement		Male	Female	Test statistic	p-value	
Mean ± SD		0.36 ± 0.48	0.75 ± 0.66	88416.00	< 0.001*	
Median (IQR)		0.00 (1.00)	1.00 (1.00)			
**Associations between pain score and obturation technique.**						
Measurement		CLC	*CWC*	SC	Test statistic	p-value
Mean ± SD		0.56 ± 0.61^A^	0.65 ± 0.64^A^	0.54 ± 0.61^A^	4.18	0.124
Median (IQR)		0.50 (1.00) ^A^	1.00 (1.00) ^A^	0.00 (1.00) ^A^		
**Associations between pain score and sealer type.**						
Measurement		CS		AH Plus	Test statistic	p-value
Mean ± SD		0.53 ± 0.62		0.69 ± 0.61	77364.00	< 0.001*
Median (IQR)		0.00 (1.00)		1.00 (1.00)		
**Associations between pain score for the two used sealer types across different obturation technique.**						
Measurement		CLC	*CWC*	SC	Test statistic	p-value
CS	Mean ± SD	0.54 ± 0.67^A^	0.50 ± 0.58^A^	0.54 ± 0.61^A^	0.23	0.890
	Median (IQR)	0.00 (1.00) ^A^	0.00 (1.00) ^A^	0.00 (1.00) ^A^		
AH Plus	Mean ± SD	0.58 ± 0.53	0.80 ± 0.67	NA	13108.50	0.010*
	Median (IQR)	1.00 (1.00)	1.00 (1.00)	NA		
Test statistic		12069.00	13927.50	NA		
p-value		0.330	< 0.001*	NA		
**Associations between pain score and sealer extrusion regardless the type of sealer**						
Measurement		Sealer extrusion (no)		Sealer extrusion (yes)	Test statistic	p-value
Mean ± SD		0.52 ± 0.61		0.67 ± 0.62	78930.00	< 0.001*
Median (IQR)		0.00 (1.00)		1.00 (1.00)		
**Associations between pain score and sealer extrusion with each sealer type**						
Measurement		Sealer extrusion (no)		Sealer extrusion (yes)	Test statistic	p-value
CS	Mean ± SD	0.45 ± 0.62		0.60 ± 0.61	28777.50	0.004*
	Median (IQR)	0.00 (1.00)		1.00 (1.00)		
AH Plus	Mean ± SD	0.62 ± 0.59		0.78 ± 0.63	12577.50	0.030*
	Median (IQR)	1.00 (1.00)		1.00 (1.00)		

Values with **different superscripts** within the **same horizontal row** are significantly different

* Significant (*p* < 0.05)

NA Not Applicable

## Discussion

Over time, a wide range of formulations for root canal sealers have been developed. Materials’ formula has a direct impact on their chemical reactions and characteristics. Among the root canal sealers examined in this study, there was a significant difference in the postobturation pain results between the root canal sealers, so the null hypothesis was rejected.

In our study using CWC-AH was associated with significantly higher pain intensity over than CWC-CS and CLC-AH Plus, concluded that both obturation technique and sealer types may be an influencing factor for initiating postoperative pain. These results align with some previous studies comparing the effects of both sealer types and obturation techniques on the incidence and intensity of postoperative pain but in contrast with others, and this could be mainly related to the selected treatment-protocol used in these studies. Our results are in agreement with the findings obtained by Koçer et al. [2] how studied the effect of obturation techniques mainly CLC, thermoplasticised solid-core carrier (GuttaCore) based filling and cold free-flow compaction (GuttaFlow2) technique on postoperative pain, his findings showed that with the exception of 12 h, there were significant differences between the groups during the first 4 days (*p* < 0.05), Bugea et al. [3] evaluated the postoperative pain after using different endodontic obturation techniques and two types of sealer, in his study he compared between warm vertical compaction with gutta-percha and zinc oxide eugenol, single cone technique used with bioceramic sealer, thermoplastic guttapercha injection with bioceramic sealer (bioconeless technique), and thermoplasticized gutta-percha injection with zinc-oxide-eugenol sealer (coneless technique), his study reported a significantly higher value of analgesic intake for the warm vertical compaction technique; additionally, the coneless technique was linked to a significantly higher analgesic intake than the single cone and bioconeless methods, and Khandelwal et al. [13] studied the postoperative pain after root canal treatment using three different base endodontic sealers Tubli-Seal, AH Plus and BioRoot RCS, his results reported that BioRoot RCS was associated with less postoperative pain than with AH Plus and Tubli-Seal sealer.

On other hand, Atav Ates et al. [14] evaluated the incidence of postoperative pain after using iRoot SP and AH Plus sealer with carrier-based obturation system, they reported that there was no significant difference between the studied groups regarding the incidence of postoperative pain. Ferreira et al. [15] compared the incidence and intensity of postoperative pain and the rate of analgesic intake after root canal treatment using AH Plus, Endofill and MTA Fillapex root canal sealer, his results showed that at any timepoint, there were no significant differences between the studied groups in the incidence or severity of postoperative pain or the need to use analgesics. Fonseca et al. [16] evaluated the intensity of postoperative pain after endodontic treatment using bioceramic and a resin-based endodontic sealer, his results showed that in both groups, the mean number of analgesic intake and the average level of pain were similar in both groups. Song et al. [17] evaluated the sealer-based root canal obturation technique using calcium-silicate-based sealer and epoxy-resin-based sealer AH Plus, ADseal, CeraSeal, and EndoSeal TCS regarding the short-term clinical effectiveness, this study showed that there was no significant difference between studied groups regarding the sealer extrusion, and regardless of sealer type, postoperative pain was not significantly indicated. Finally **Seron** et al. [18] in his systematic review and meta-analysis assessed the postoperative pain after root canal treatment using both bioceramic and AH plus sealer, The findings demonstrated that bioceramic sealers decreased postoperative endodontic pain only after a 24-hour period.

Our finding showed that bioceramic sealer was associated with lower postoperative pain than resin-based sealer regardless the obturation technique used, this result is in agreement with other studies finding [18, 19], however other studies did not report a difference between the bioceramic- and resin- based root canal sealers [14–16, 20, 21], on other hand, other studies showed that bioceramic sealer was associated with higher postoperative pain than resin-based sealer [22], the difference of these results may be attributed the heterogeneity of the studies and different inclusion parameters [17, 20].

The severity and intensity of postoperative pain are significantly influenced by the composition of the root canal sealer due to the release of chemical irritants during setting reaction that may cause local inflammation. Higher incidence of postoperative pain in the case of AH Plus sealer obtained in our study, could be explained by its cytotoxicity as it release toxic monomers such epoxy resin and bisphenol A diglycidyl ether [23, 24], also presence of unpolymerized residues which formed due to the ether development of an oxygen inhibition layer in the sealer mixture [19, 21]. Additionally, the AH Plus sealer’s 7-hour delayed setting time raises concerns about biocompatibility and may result in the release of cytotoxic components prior to setting, which leads to more frequent pain episodes [19]. However, a negative tissue reaction, like an inflammatory or foreign body reaction, could be triggered by the type of sealer utilized [25], other negative reaction may be in the form of sensory nerve activation, however this is an uncommon event [26]. The the properties of AH plus sealer undergoes modifications when heat is applied during CWC technique, the amine groups which act as setting initiators in AH plus are lost when heated to 100 °C for 1 min leading to retardation of polymerization reaction, reduction in the setting time, and increase in film thickness [27]. Bioceramic based sealer has the advantage of having biocompatible ingredients without resin, which is supported by Riccuci et al., who showed that the histologic section that extruded bioceramic based sealer has no foreign body or inflammatory response [28]. Several biological advantages of bioceramic based sealer may be related to its high pH which might encouraging the creation of hard tissue and preventing osteoclastic activity, which could result in favorable healing [29].

Our study found no statistically significant difference between groups regarding the incidence of sealer extrusion. However, it’s worth noting that sealer extrusion was observed more frequently in the SC-CS groups compared other groups. This finding is interesting in light of previous research suggesting that bioceramic sealers like CeraSeal are more biocompatible and less likely to cause an inflammatory response when in contact with periapical tissues [5]. Despite the higher incidence of extrusion, this did not translate into significantly higher pain scores for the CeraSeal groups, which may support the claimed biocompatibility of these materials. Our finding showed no significant different between the incidence of the sealer extrusion and the type of sealer used with high rate related to CeraSeal group, this result are in contrast to results shown by Fonseca et al. [16] who showed that bioceramic group sealer had significantly more extrusion incidence than the resin group sealer, this may be attributed to the selection of single-rooted maxillary teeth that prepared up to #40 and 6% taper single-file reciprocating system, Seron et al. [18] in his systematic review and meta-analysis reported that bioceramic sealer showed less sealer extrusion compared to the AH Plus, in this meta-analysis study five studies were performed with multirooted teeth and seven studies used both tooth morphologies. Zamparini et al. [17] in his recently published systematic review and meta-analysis concluded that premixed bioceramic sealers’ apical extrusion ranged from 11.8 to 59.8%, whereas controls’ ranged from 11.8 to 33.3%. The incidence of sealer extrusion seems to be more affected by the operator, root canal preparation, and the obturation techniques utilized than the type of root canal sealer selected [21].

The lower pain scores result from our study is mainly related to treatment-parameters and inclusion criteria selected in the study, a significant finding of our study was the evolution of pain over time within all groups. Generally, pain scores were highest at 6 h post-treatment, gradually decreasing over the following time periods. This pattern was consistent across all groups, regardless of the obturation technique or sealer used. This pain pattern aligns with the findings of other studies [2, 17, 20, 21, 30], who reported that postoperative pain is most intense in the immediate aftermath of treatment and gradually subsides over time. The decrease in pain intensity over time is likely due to the resolution of acute inflammatory responses triggered by the endodontic procedure.

### Clinical implications

The findings of this study have several important clinical implications. Given the similar pain profiles across different techniques, clinicians may have more flexibility in choosing an obturation method based on other factors such as case complexity, time constraints, or personal preference. Additionally, the comparable performance of bioceramic and resin-based sealers in terms of postoperative pain suggests that both are viable options. The choice may then depend on other factors such as long-term sealing ability or ease of retreatment. The consistent pattern of pain evolution across all groups can help in setting realistic expectations for patients. Clinicians can confidently inform patients that they may experience moderate pain in the first 24 h, which should significantly decrease over the following days. Moreover, the overall low to moderate pain scores observed support the viability of single-visit endodontic treatments, even for multirooted teeth with asymptomatic irreversible pulpitis.

### Limitations and future directions

While this study provides valuable insights, it has some limitations. The follow-up period was relatively short (72 h), and long-term outcomes were not assessed. Future studies could extend the follow-up period to evaluate long-term pain patterns and treatment success rates. Additionally, while we controlled factors such as preoperative pain and systemic health conditions, other variables such as operator experience, precise canal anatomy, or patient pain thresholds could influence outcomes. Multi-center studies with larger sample sizes could help account for these variables. Future research could also explore the correlation between sealer extrusion and postoperative pain in more detail, perhaps using 3D imaging techniques to quantify the volume of extruded material accurately.

## Data Availability

No datasets were generated or analysed during the current study.

## Footnotes

1 R Core Team (2024). R: A language and environment for statistical computing. R Foundation for Statistical Computing, Vienna, Austria. URL https://www.R-project.org/.
